# Short-course, oral flubendazole does not mediate significant efficacy against *Onchocerca* adult male worms or *Brugia* microfilariae in murine infection models

**DOI:** 10.1371/journal.pntd.0006356

**Published:** 2019-01-16

**Authors:** Hanna T. Sjoberg, Nicolas Pionnier, Ghaith Aljayyoussi, Haelly M. Metuge, Abdel J. Njouendou, Valerine C. Chunda, Fanny F. Fombad, Dizzle B. Tayong, Narcisse V. T. Gandjui, Desmond N. Akumtoh, Patrick W. N. Chounna, Bertrand L. Ndzeshang, Sophie Lachaud, Fetene Tekle, Ludo Quirynen, Marc Engelen, Benny Baeten, Andrew Steven, Stephen A. Ward, Mark J. Taylor, Samuel Wanji, Joseph D. Turner

**Affiliations:** 1 Department of Parasitology and Research Centre for Drugs and Diagnostics, Liverpool School of Tropical Medicine, Pembroke Place, Liverpool, United Kingdom; 2 Research Foundation in Tropical Medicine and the Environment, Buea, Cameroon; 3 Department of Microbiology and Parasitology, University of Buea, Buea, Cameroon; 4 Janssen Pharmaceutica, Beerse, Belgium; Uniformed Services University of the Health Sciences, UNITED STATES

## Abstract

The *Onchocerca ochengi* adult implant and *Brugia malayi* microfilariemic Severe-Combined Immunodeficient (SCID) mouse models are validated screens to measure macrofilaricidal and microfilaricidal activities of candidate onchocerciasis drugs. The purpose of this study was to assess whether 5 daily sub-cutaneous (s.c.) injections of standard flubendazole (FBZ) suspension (10mg/kg), a single s.c. injection (10mg/kg) or 5 daily repeated oral doses of FBZ amorphous solid dispersion (ASD) formulation (0.2, 1.5 or 15mg/kg) mediated macrofilaricidal efficacy against *O*. *ochengi* male worms implanted into SCID mice. The direct microfilaricidal activity against circulating *B*. *malayi* microfilariae of single dose FBZ ASD formulation (2 or 40 mg/kg) was also evaluated and compared against the standard microfilaricide, ivermectin (IVM). Systemic exposures of FBZ/FBZ metabolites achieved following dosing were measured by pharmacokinetic (PK) bioanalysis. At necropsy, five weeks following start of FBZ SC injections, there were significant reductions in burdens of motile *O*. *ochengi* worms following multiple injections (93%) or single injection (82%). Further, significant proportions of mice dosed following multiple injections (5/6; 83%) or single injection (6/10; 60%) were infection negative (drug-cured). In comparison, no significant reduction in recovery of motile adult *O*. *ochengi* adult worms was obtained in any multiple-oral dosage group. Single oral-dosed FBZ did not mediate any significant microfilaricidal activity against circulating *B*. *malayi* mf at 2 or 7 days compared with >80% efficacy of single dose IVM. In conclusion, multiple oral FBZ formulation doses, whilst achieving substantial bioavailability, do not emulate the efficacy delivered by the parenteral route *in vivo* against adult *O*. *ochengi*. PK analysis determined FBZ efficacy was related to sustained systemic drug levels rather than achievable Cmax. PK modelling predicted that oral FBZ would have to be given at low dose for up to 5 weeks in the mouse model to achieve a matching efficacious exposure profile.

## Author summary

Onchocerciasis, caused by the filarial parasitic worm, *Onchocerca volvulus*, is a major cause of infection-related blindness and skin disease and is targeted for elimination. Current drugs are not optimal in all patient populations and a short-course cure would accelerate elimination. Flubendazole is used to treat intestinal worms in humans. The current oral formulation is not well absorbed into the blood. Flubendazole treatment has mediated curative efficacy in onchocerciasis patients when given as an injection but also causes adverse reactions at the injection site. In this study, we tested whether a new oral formulation of flubendazole with improved absorption could selectively kill *Onchocerca* adult parasites compared with circulating larval filarial parasites, using immunodeficient mouse models. Whilst injections of flubendazole mediated high levels of efficacy, five-day oral treatment, at a range of doses, did not emulate this curative effect. Oral flubendazole also did not significantly affect levels of microfilariae in the blood. After comparing levels of flubendazole in the blood following injection versus oral treatment, we conclude that a long duration, low dose of the oral formulation, for 5 weeks, would be needed to match the exposure of the drug following injection.

## Introduction

Onchocerciasis remains a severe public health problem despite the sustained efforts of mass drug administration (MDA) programs aimed at eliminating this vector-borne, parasitic neglected tropical disease [1–3]. Elicited by the filarial nematode *Onchocerca volvulus* and transmitted by black flies of the genus *Simulium*, it is endemic in much of Sub-Saharan Africa, as well as more limited foci in Brazil, Venezuela, and The Yemen with 37 million people infected [4, 5]. The pathology associated with onchocerciasis ranges from troublesome skin itching, skin disease (onchodermatitis), to blindness, which is caused by a sclerosing ocular keratitis that affects 0.8 million individuals and is the second cause of infectious blindness after trachoma [5–7]. Onchocercal disease is induced by death of migratory larval microfilariae (mf), causing liberation of inflammatory stimuli including the endosymbiont *Wolbachia* and inflammatory recruitment of granulocytes [8, 9]. The debilitating symptoms of onchodermatitis and river blindness also cause great economic losses in endemic areas [10].

Onchocerciasis is targeted for elimination as a public health problem [11, 12]. The African Program for Onchocerciasis Control (APOC) and, latterly, The Extended Special Project for Elimination of NTDs (ESPEN) were created to this end [13]. The current strategy involves the use of the microfilaricidal drug, ivermectin (IVM, Mectizan). Ivermectin targets mf produced by mating adult filariae, and is deployed using an MDA strategy [2, 3, 14, 15].

Because IVM is microfilaricidal yet lacks significant macrofilaricidal activity, it must be administered repetitively and with very high population coverage over an extended period of time in order to break transmission. This is predicted as at least twelve annual treatment rounds [2, 3, 16].

The undeniable success of MDA programs can be seen in certain country settings where a decline in prevalence has been recorded as well as a reduction in disease burden [17]. However, in other countries, the limitation of MDA programs for onchocerciasis within Africa is evident and several persistent areas of infection remain, possibly due to emerging resistance [18–21]. Also, in certain regions, poor adherence to IVM treatment is apparent and associated with sustained skin infection prevalence. Where onchocerciasis MDA overlaps with health districts endemic for the related filaria, *Loa loa*, low adherence to treatment is linked to the perceived risk of IVM induced severe neurological adverse reactions [22, 23]. Therefore, if onchocerciasis elimination targets within the ambitious 2030 United Nations Sustainable Development Goal timeframes are to be achieved, there is an urgent need to implement alternative strategies.

For these reasons, a safe, chemotherapeutic agent that will selectively kill adult *O*. *volvulus* worms without mediating rapid ‘ivermectin-like’ microflaricidal activity, is urgently needed, preferably with a short treatment period of less than 7 days. Typically, the most practical and safest administration of a field agent is through oral dosage [24].

Flubendazole (FBZ), is a benzimidazole anthelmintic that acts through interfering with the equilibrium among tubulin subunits and elicits macrofilaricidal effects by preferentially binding to nematode tubulin [25]. FBZ was initially developed for use against gastrointestinal parasitic nematodes in livestock [26], but was later approved for use in humans in the treatment of soil-transmitted helminths with high efficacy [27–29]. The drug has also been shown to be highly efficacious in experimental models of lymphatic filariasis when administered subcutaneously [24, 30, 31] as well as in a human trial treating onchocerciasis [32]. *Onchocerca ochengi*, a cattle parasite, is the closest phylogenetic relative to the target human parasite, *O*. *volvulus*. Recently, multiple subcutaneous injections of FBZ has mediated >90% efficacy against *O*. *ochengi* in severe-combined immunodeficient (SCID) mice [33]. Unfortunately, the approved formulation has a very limited bioavailability when orally administered. When administered parenterally, severe reactions around the subcutaneous injection site were reported in the clinical trial [32].

Efforts have been made to develop a re-formulation of FBZ that would enable oral dosing [24, 34, 35]. The purpose of this study was to assess the oral efficacy of a new formulation of FBZ (Janssen Pharmaceutica) in SCID mice implanted with adult male *O*. *ochengi* parasites. Single versus multiple subcutaneous doses of standard FBZ suspension were also compared. In addition, the selective toxicity of oral FBZ against adult *Onchocerca* versus bloodborne mf in circulation was tested in a *Brugia malayi* SCID mouse infusion model. The primary goal was to identify regimens that cause minimally 90% reduction in adult *Onchocerca* parasite numbers in treated animals compared to untreated controls.

## Methods

### Animals

Male CB.17 (BALB/c congenic) SCID mice, 5–6 weeks of age, were purchased from Charles River Europe. Mice were shipped to either The Research Foundation for Tropical Diseases and the Environment (REFOTDE), Buea, Cameroon (for *O*. *ochengi* studies) or University of Liverpool Biological Services Unit (UoL BSU), UK (for the *B*. *malayi* study) in filter topped boxes. Mice were maintained in individually ventilated caging (IVC) with HEPA filtered air system (Tecniplast, UK) with autoclaved bedding and fed / watered *ad libitum* with UV sterilised appropriate certified rodent diet (irradiated chow) / autoclaved water. Following a 7 days acclimatization period, SCID mice were 7–8 weeks of age at initiation of studies.

Mongolian gerbils were originally purchased from Charles River and a colony bred at UoL BSU under specific-pathogen-free (SPF) conditions.

### Ethics statement

All experiments carried out in Cameroon were approved by the Animal Care Committee, REFOTDE. All experiments undertaken in UK were approved by institutional Animal Welfare Ethics Review Boards of University of Liverpool and Liverpool School of Tropical Medicine and were undertaken in accordance with national legislation (Home Office Project Licence 30/2974).

### Isolation of male *O*. *ochengi*

*O*. *ochengi* positive cattle from Adamawa Province, Cameroon, were selected by nodule palpation in the umbilical region. The *O*. *ochengi* positive cattle were transported to the South West Province and maintained in pastureland local to REFOTDE, Buea. Individual infected cattle were slaughtered at a local abattoir. For each cow, the umbilical region was excised and transported to REFOTDE within 2 hours after slaughter. Skin samples were washed and rinsed in sterile PBS, the hair removed by shaving and onchocercomata (nodules) were excised from the dermal surface using sterile scalpels and forceps. Nodules were immediately placed in complete sterile RPMI medium (10% foetal calf serum, 100U/ml penicillin, 0.1mg/ml streptomycin and 2.5*μ*g/ml amphotericin B), the capsular surface of the nodule incised and gentle pressure applied to liberate adult stage *O*. *ochengi* parasites. Nodules were incubated in petri dishes in complete medium for 4h, 37°C, 5% CO_2_ to allow adult male parasites to migrate from nodule tissue. Intact, motile male *O*. *ochengi* parasites were washed in sterile complete RPMI and incubated overnight 37°C, 5% CO_2._

### Experimental implantation with *O*. *ochengi*

SCID mice were weighed and ear marked, anaesthetised by a combination of ketamine and medetomidine (1/10*μ*g/kg subcutaneous (SC)), administered with prophylactic antibiotic penicillin G, SC, shaved on the upper left abdomen and swabbed with iodine. Anaesthetised mice were arranged on sterile drapes on top of heat pads, and a small incision made through both skin and abdominal wall with sterile surgical instruments. Fifteen motile *O*. *ochengi* male worms were picked from cultures using sterile forceps and implanted into the peritoneal cavity with 0.5ml RPMI. Efficiency of implantation was confirmed by verifying absence of male worms in the dish and on forceps by washing with medium. Mice were sutured through the abdomen and skin and iodine re-applied, administered with an α2-antagonist (atipamezole) and placed on heat pads until recovered. Post-surgery and recovery from anaesthesia, mice were housed in original family groups in IVCs, monitored regularly.

### *B*. *malayi* microfilarial purification and experimental infection

*B*. *malayi* microfilariae (*Bm*mf) were isolated from the peritoneal cavity of experimentally infected Mongolian gerbils by peritoneal washing in RPMI medium containing 100U/ml penicillin, 0.1mg/ml streptomycin (RPMI+PS) using a sterile catheter drain, under anaesthesia, as previously described [33]. Motile *Bm*mf were purified from host cells by PD-10 desalting sephadex column (GE Healthcare) at room temperature (rt), washed 3x in RPMI+P/S rt, resuspended in known volume of RPMI+PS, maintained at 37°C, 5%CO_2_ whilst density was enumerated by microscopy. Motile *Bm*mf were adjusted to 1.25x10^6^/ml in RPMI+PS 37°C. Subsequently, 200μl aliquots were loaded into 1ml low dead space syringes with 29 gauge needles and maintained at 37°C.

### *B*. *malayi* microfilarial experimental infection

Mice were placed in small animal thermal cages at 37°C for 5 minutes to dilate the tail vein. Motile *Bm*mf were infused via the lateral tail vein with manual restraint in a maximum volume of 200μl. Efficiency of inoculation was confirmed by needle washout and enumeration of mf retained in the needle / syringe. Efficiency of inoculation ((inoculation number—needle washout number) / inoculation number x100) was >96% in all animals infused.

### Drug preparation

All compounds and vehicles were provided by Janssen and the formulations were prepared on site at REFOTDE, Buea or shipped prepared to Liverpool. The vehicle for FBZ Bend 1/9 oral (OR) suspensions (JNJ-161941-AAA/HPMC AS-HG 1/9) was 0.5% w/v Methocel A4M (Premium) in demineralized water. The vehicle for the FBZ SC suspension was 0.5% w/v HEC (Hydroxyethylcellulose) in demineralized water + 0.1% Tween80. Briefly, oral FBZ suspensions were prepared from the supplied Spray Dried Drug and first homogenised in demineralised water using a Polytron disperser then made up to volume with Methocel, a different formulation was prepared for each treatment group to keep the dosing volume constant. The subcutaneous suspension was first homogenised in demineralised water + 0.1% Tween80 using a Polytron disperser then made up to volume with HEC.

### Drug treatment randomisation and blinding

Following surgical implantation with *O*. *ochengi* or infusion with *Bm*mf, mice were ear-notched and individuals involved in dosing assigned mice a unique identification code and randomly assigned mice to test or control groups. A sample size of n = 11 for test groups was determined as minimally adequate to detect a ≥90% average reduction in adult male *O*. *ochengi* worm burden with >80% power, based on prior variation in worm burden recovery rate in untreated or vehicle dosed SCID mice (mean % recovery = 29.07, s.d. = 20.1) [33, 36]. A group size of n = 5 for test groups was determined as minimally adequate to detect ≥70% average reduction in circulating *Bm*mf 2 days following treatment based on prior variation in microfilaraemias per millilitre of tail vein blood in vehicle dosed SCID mice (mean mf/ml = 242, s.d. = 225) [33]. *O*. *ochengi* implanted mice from an individual donor source (each individual cow) were distributed evenly within each treatment / control group to minimize bias due to inter-donor variation and/or experimental variation in daily isolations potentially affecting long-term adult *O*. *ochengi* viability. For *O*. *ochengi* testing, two experiments (A and B) were conducted. In experiment A there was one untreated (negative) control group (n = 11) and a positive control group was included in which FBZ was administered SC by injection at 10 mg/kg once per day (QD) for 5 consecutive days (n = 6 mice). In experiment B, there was one vehicle (negative) control group (n = 11). For *B*. *malayi* testing, one vehicle (negative) control group and one positive control group was included where IVM was administered OR at 0.2 mg/kg once (both n = 5). Dosing for all experiments is listed in Table 1.

**Table 1:** Dose groups for oral FBZ testing.

EXPT	Group	n	Dosing regimen	Route	Formulation	Volume / kg	Dose (mg/kg)
Experiment A: Male *Onchocerca* implants	A1	11			untreated control		
	A2	6	QD 5x	SC	FBZ HEC/Tween	10ml	10
	A3	11	QD 5x	OR	FBZ bend 1/9	10ml	0.2
	A4	11	QD 5x	OR	FBZ bend 1/9	10ml	1.5
	A5	11	QD 1x	SC	FBZ HEC/Tween	10ml	10
Experiment B: Male *Onchocerca* implants	B1	10	QD 5x	OR	vehicle control	10 ml	0
	B2	11	QD 5x	OR	FBZ bend 1/9	10 ml	15
*B*. *malayi* mf infusions	1	5	Single oral dose	OR	Vehicle control		
	2	5	Single oral dose	OR	IVM	10ml	0.2
	3	5	Single oral dose	OR	FBZ bend 1/9	10ml	2
	4	5	Single oral dose	OR	FBZ bend 1/9	10ml	40

Individuals involved in randomisation were also involved in dosing and were thus unblinded to treatment. Individuals responsible for evaluating primary and secondary efficacy readouts were blinded as to treatment group.

### Pharmacokinetic sampling and analysis

For all treated groups blood was collected for pharmacokinetic analysis. A plasma profile was sampled on the final day of dosing (or on the day of dosing for single dose SC groups). SC groups were continuously sampled after dosing by one sample per week until necropsy. For each time point, 3 animals per group were sampled, using a micro-sampling technique. Each animal was sampled a maximum of twice per day. The exact time of sampling was recorded for each sample. Approximately 20μl blood was collected per animal from the tail vein, using EDTA-coated Microvette® CB 300 K2E tubes (Sarstedt). Blood was placed immediately on ice prior to centrifugation. After centrifugation (1900*g for 10 min) 10μl plasma was transferred into a 0.5 ml Eppendorf tube and placed immediately at -20°C. All plasma samples were shipped on dry ice to Janssen Beerse via Liverpool School of Tropical Medicine in a single batch at the end of the study. Analysis of the plasma samples was done at PD&S-PDM regulated bioanalysis department (J&J PRD, Beerse). Plasma samples were analysed for JNJ-161941 (FBZ) and the metabolites JNJ-1809600 (R-FBZ) and JNJ-114699 (H-FBZ) using a qualified LC-MS/MS method. The following pharmacokinetic parameters were calculated: C_max_, T_max_, and AUC values. Dose-proportionality was evaluated. The plasma curves were prepared in the same matrix as the plasma study samples. For each analytical run QC samples were analysed together with the study samples and calibration curve. All analytical batches were accepted based on calibration curve and QC acceptance criteria in line with the current FDA guidelines. A limited pharmacokinetic analysis on the mean plasma concentrations was performed using Phoenix™ Professional (Version 6.2.1). A non-compartmental analysis using the lin/log trapezoidal rule with lin/log interpolation was used for all data.

For simulating PK profiles of different dosing regimes, the PK data was fitted using a two compartment model for the SC formulations and a one compartment model for OR preparations. PK parameters were generated for each experimental arm using ADAPT 5 (Biomedical Simulations Resource–University of California). The PK parameters were then then used to generate simulations for different dosing scenarios using the ADAPT 5 simulator. For PK analysis SC and ORl data were fitted using the following equations.

$$\frac{dX_{1}}{dt}=-k_{a}∙X_{1}$$

$$\frac{dX_{2}}{dt}=k_{a}∙X_{1}-(k_{cp}+k_{e})∙X_{2}+k_{pc}∙X_{3}$$

$$\frac{dX_{3}}{dt}=k_{cp}∙X_{2}-k_{pc}∙X_{3}$$

$$C=\frac{X_{2}}{V}$$

Where ***X***_***1***_ represents the drug mass in dosing compartment, ***X***_***2***_ represents drug mass in the systemic circulation and ***X***_***3***_ represents the drug mass peripheral compartment. ***k***_***a***_ represents the rate of absorption (*h*^*-1*^), ***k***_***e***_ the rate of drug elimination (*h*^*-1*^), ***k***_***cp***_ and ***k***_***pc***_ the rates of transfer between the central and peripheral compartments (*h*^*-1*^), ***V*** represents the volume of distribution (*mL*) and ***C*** is the concentration of drug at any given time (*mg/L*).

For the OR preparations, a one compartment model was sufficient to fit the data as evidenced by AIC and BIC parameters as well as the intercompartmental transfer rates being negligible. For these reasons, the parameters *k*_*cp*_ and *k*_*pc*_ were fixed to zero to fit the oral data into a one compartment model.

### Adult *O*. *ochengi* parasite recovery and motility assay post-treatment

Mice were euthanized by UK Home Office approved schedule 1 method at 5 weeks post-first dose administration. The primary efficacy parameter was the worm burden of live male worms recovered after necropsy from the abdominal cavity, including visceral connective tissues. As secondary readout, cure rate (number of mice without active infection) was also evaluated. As a further secondary readout, motility scoring of recovered adult male *O*. *ochengi* parasites was determined by visual inspection after 15 minutes in culture in 37°C RPMI post-recovery, including motility in response to gentle prodding with a blunt pipette. A semi-quantitative score for worm motility was applied. Worms that showed no motility were counted as ‘0’ = immotile, ‘1’ = only anterior or posterior twitching motility ‘2’ = reduced sigmoidal motility ‘3’ = full sigmoidal motility.

### MTT-formazan reduction colourmetric assay

As an additional secondary readout, male *O*. *ochengi* recovered at necropsy were washed in PBS and individually placed in a solution of MTT in PBS, final concentration 0.5 mg/ml, incubated for 2 hours at 37°C with 5% CO_2_. After washing in PBS, the *O*. *ochengi* were incubated in 100% DMSO for 1 hour at 37°C with 5% CO_2_ to dissolve and release the blue formazan product. The samples were read at OD 490 nm.

### *B*. *malayi* microfilaraemia quantifications

For primary efficacy readout the number of circulating mf were enumerated by tail vein scratch sampling method and collection of 2x20μl fresh blood aspirated by Gilson pipette at baseline and 2 days post-treatment. For secondary readout, mice were euthanized by UK Home Office approved schedule 1 method at 7 days post-treatment. A 40μl blood volume was collected by cardiac puncture immediately after cullling. Immediately after blood collections, volumes were spread onto uncoated glass slides to make air-dried thick smear preparations. Briefly blood was collected either from the tail vein (2x20*μ*L, as for peripheral blood microfilaremia) or from the heart (2x30*μ*L at necropsy using a 25G 1mL syringe, as for cardiopulmonary microfilaremia) and transferred onto a glass slide and then processed for a thick smear through a scratch method. Resulting smear was left to dry then slides were incubated in distilled water for 4min to lyse erythrocytes, fixed in methanol for 1min and finally stained with 40% Giemsa for 40min followed by a wash in distilled water until clear. Total numbers of *Bm*mf were counted per two replicate slides by microscopy and adjusted (x25) to obtain a parasitaemia per millilitre of blood. Of the 120 thick blood smears evaluated, 10% (n = 12) were independently re-assessed by a scientist not directly involved in the study and percentage variance was determined as <20% for all samples (mean = 10.3%,+/-5.59% S.D.).

### Statistical analysis

For primary readout analysis of *O*. *ochengi* worm recovery, a dose/schedule providing a reduction in average worm burden of ≥90% versus the control group was considered as efficacious.

The percent effectiveness was calculated using geometric mean (GM) as follows:

$$\%efficacy=\frac{\mathrm{GM}\;untreated\;group-GM\;dose\;group}{GM\;untreated\;group}X100,$$

where GM is a geometric mean of the number of male *O*. *ochengi* recovered at 5 weeks post-treatment for each group. Since the number of recovered male *O*. *ochengi* at 5 weeks post-treatment was zero for some animals, a value of 0.1 was added to each data for the calculation of the GM. Number of male *O*. *ochengi* recovered at 5 weeks post-treatment (+0.1) for both experiments were examined for normality, using the Shapiro-Wilk test [37]. The data failed to pass this test. Therefore, data was log10 transformed and the transformed data was tested for normality as before. The log10 transformed data did not satisfy the Shapiro-Wilk test for normality. Therefore, Kruskal-Wallis non-parametric test was used to compare the groups in experiment A and followed by post-hoc Dunn’s test [38] to compare each dose group to the untreated group. The Mann Whitney non-parametric test was used on the original raw data to compare the two groups in experiment B.

For primary readout analysis of *Bm*mf in the peripheral circulation, a dose/schedule providing a reduction in average worm burden of ≥70% versus the vehicle control group 2 days after dosing was considered as efficacious as a rapid microfilaricide. The efficacy of a test group was calculated as following:

$$\%effectiveness=\frac{100\;\left(GM\;microfilariaemia\;baseline–GM\;microfilariaemia+48h\right)}{GM\;microfilariaemia\;baseline}$$

For changes in peripheral microfilaraemias, change was calculated as follows:

deltaperipheralmf/ml=mf/mlat+2daysor+7days–mf/mlbaseline.

Delta peripheral mf/ml at +2 day and cardiopulmonary circulating mf/ml +7 days post-treatment continuous variable distributions were examined for normality by the Shapiro-Wilk test. Data that was not normally distributed was then log10 transformed before being re-tested for normality. All raw data or log10-transformed data did not deviate from a normal distribution pattern. Therefore, 1 Way ANOVA with Dunnett’s tests were applied to examine significant differences between vehicle and drug groups in delta peripheral mf/ml at +2 days and Log10 cardiac mf/ml at +7 days post-treatment. All tests were performed at a significance level of 5%.

Additional efficacy parameters analysed in both *O*. *ochengi* experiment A and B were: 1. frequency of animals with zero worm count / group (cure rate) 2. the frequency of mice with normal vs reduced motile male *O*. *ochengi* present at necropsy 3. the metabolic activity of motile male *O*. *ochengi* retrieved at necropsy. The proportions of mice with or without infection were expressed as percentages of the total group number. Raw data (numbers of infected and uninfected mice) were compared for each group in a 5x2 contingency table and tested for significance by Chi-square Test. Post-hoc tests were undertaken comparing control against specific treatment groups by 2x2 contingency tables and two-tailed Fisher’s Exact Tests. For *O*. *ochengi* metabolic activity, formazan optical densities from worm extractions were averaged (mean) per mouse in each group, measured by MTT-formazan reduction colourmetric assay. Mean *O*. *ochengi* metabolic activity data was checked for normality, using the Shapiro-Wilk test. Subsequently, 1 Way ANOVA with post-hoc Dunnett’s Tests were used to determine significance between control and treated groups of mice. All tests were performed at a significance level of 5%.

## Results

### Assessment of *O*. *ochengi* worm burden 5 weeks post-dosing with FBZ

**Fig 1:**
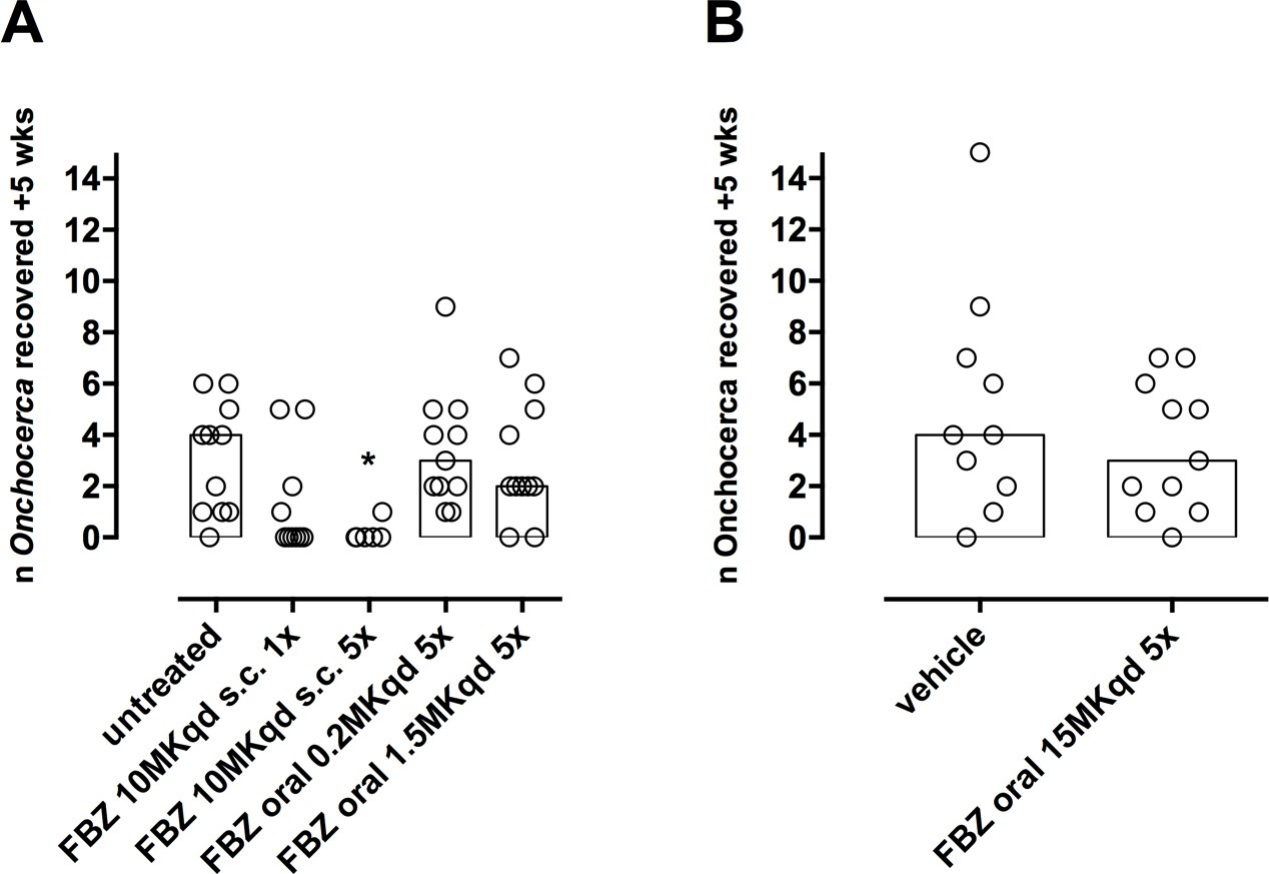
*O*. *ochengi* male worm burdens 5 weeks following indicated treatments for experiments (A) and (B). Bars are median recovered numbers per group. Treatment with s.c. injected FBZ 10mg/kg qd 5x mediated a significant reduction in the retrieval of viable *O*. *ochengi* worms (Kruskal-Wallis ANOVA = 15.45, Dunn’s tests untreated *vs* FBZ 10mg/kg qd 5x *P*<0.05*).

Raw data for numbers of *O*. *ochengi* recovered per mouse / treatment are plotted in Fig 1 for both experiment A and B. Table 2 details the geometric means (95% confidence intervals) of raw data +0.1 and the derived calculated percentage efficacy for both experiments.

**Table 2:** *O*. *ochengi* treatment efficacy post-FBZ dosing.

Group	Treatment	n	Geometric mean +5 weeks (95% CI)	% efficacy
A1*	untreated	11	2.11 (1.74–4.65)	-
A2	FBZ 10mg/kg QD 1x SC	10	0.38 (0–2.87)	82.0
**A3**	**FBZ 10mg/kg QD 5x SC**	**6**	**0.15 (0–0.70)**	**92.9**
A4	FBZ 0.2mg/kg QD 5x OR	11	2.93 (1.98–5.13)	0
A5	FBZ 1.5mg/kg QD 5x OR	11	1.71 (1.46–4.55)	19.0
B1*	vehicle	10	5.2 (3.8–6.6)	-
B2	FBZ 15mg/kg QD 5x OR	11	3.65 (2.88–4.41)	29.9

*A = *O*. *ochengi* adult implant experiment A, B = *O*. *ochengi* adult implant experiment B

Multiple injected FBZ (10mg/kg) mediated a high level of efficacy against male *O*. *ochengi* (92.9%) and single injection (10mg/kg) also mediated substantial efficacy (82%). Oral dosing of FBZ did not emulate the efficacy of injections (0%, 19% and 29.9% for 0.2, 1.5 and 15mg/kg, respectively) (Table 2). Subsequently, the statistical variation between numbers of male *O*. *ochengi* recovered at 5 weeks post-dosing was scrutinised (Fig 1). Multiple injected FBZ (10mg/kg) significantly reduced worm burden compared with untreated controls (Fig 1A). Variation in worm burdens in all other groups were not significantly different than untreated controls (Fig 1A & 1B).

### Frequency of mice with male O. ochengi present five weeks post-dosing with FBZ

Table 3 details the proportions of mice in each dosing group that did not contain viable male *O*. *ochengi* at necropsy (cure rate) for both experiments.

**Table 3:** *O*. *ochengi* cure rates post-FBZ dosing.

Group	Treatment	n	n positive	n negative	cure rate (%)
A1*^|^	untreated	11	10	1	9.1
**A2**	**FBZ 10mg/kg**^^^ **QD 1x SC**	**10**	**4**	**6**	**60**
**A3**	**FBZ 10mg/kg**^#^ **QD 5x SC**	**6**	**1**	**5**	**83.3**
A4	FBZ 0.2mg/kg QD 5x OR	11	11	0	0
A5	FBZ 1.5mg/kg QD 5x OR	11	9	2	18.2
B1*	vehicle	10	9	1	10
B2	FBZ 15mg/kg QD 5x OR	11	10	1	9.1

*A = *O*. *ochengi* adult implant experiment A, B = *O*. *ochengi* adult implant experiment B

^**|**^Experiment A, 2x5 Chi-square, df 20.68, *P =* 0.0004***

**^**Fisher’s Exact Test (*vs* untreated), *P =* 0.0237*

^**#**^Fisher’s Exact Test (*vs* untreated), *P =* 0.0054**

### Motility assessments of male *O*. *ochengi* five weeks post-dosing with FBZ

Table 4 details the frequencies of normal or reduced motile worms five weeks post-dosing.

**Table 4:** *O*. *ochengi* motility assessments post-FBZ dosing.

Group	Treatment	n	n of animals with motile *O*. *ochengi*	Total n motile *O*. *ochengi* assessed	n *O*. *ochengi* normal/reduced motility
A1*^#^	untreated	11	10	32	21/11
A2	FBZ 10mg/kg QD 1x SC	10	4	12	10/2
A3	FBZ 10mg/kg QD 5x SC	6	1	1	0/1
A4	FBZ 0.2mg/kg 5x OR	11	11	36	30/6
A5	FBZ 1.5mg/kg QD 5x OR	11	9	29	16/13
B1*^	vehicle	10	9	47	33/14
B2	FBZ 15mg/kg QD 5x OR	11	10	38	16/22

*A = *O*. *ochengi* adult implant experiment A, B = *O*. *ochengi* adult implant experiment B

^**#**^2x5 Chi-square, df 9.73, *P =* 0.0453

^Fisher’s Exact Test (*vs* vehicle control), *P<*0.0001

In experiment A, by 2x5 Chi-square analysis, a significant difference in frequency in fully motile *vs* reduced motile *O*. *ochengi* was apparent between the groups. However, it was determined by post-hoc Fisher’s Tests, that none of the treatment groups displayed a significantly different frequency motility level when compared with the untreated controls. In experiment B, the FBZ 15mg/kg QD 5x OR regimen group displayed a significantly lower frequency of fully motile *O*. *ochengi* males compared with the vehicle control (Fisher’s Exact Test *P*<0.0001).

### Metabolic activity of motile male *O*. *ochengi* five weeks post-dosing with FBZ

**Fig 2:**
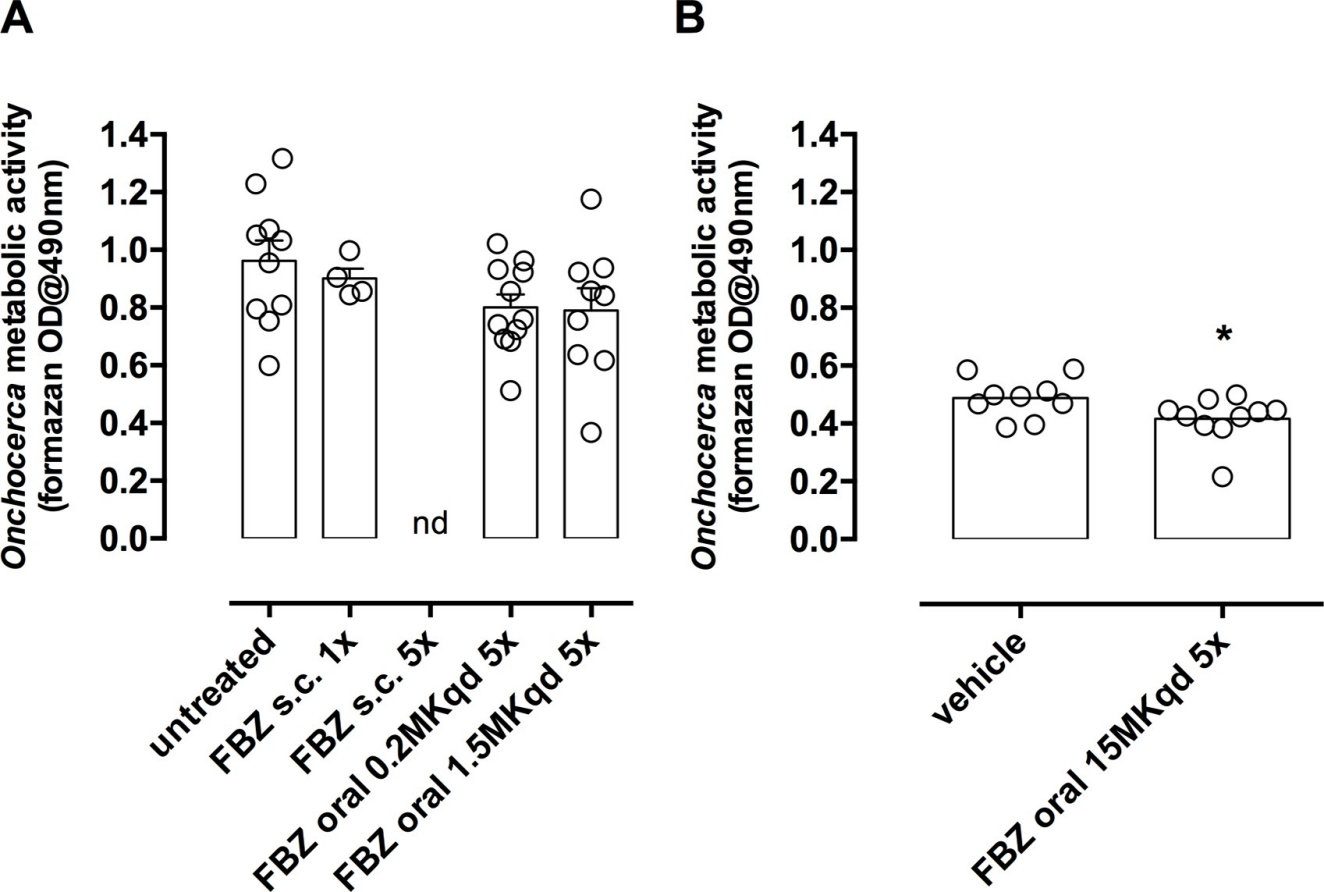
Average *O*. *ochengi* metabolic activity per mouse where motile worms were present, five weeks post-dosing with indicated treatments for experiment A (A) or B (B). Data points are mean formazan optical densities @490nm from worms recovered from each animal. Bars are mean +/-SEM per treatment group. Variation between untreated and treatment groups in experiment A was not significantly different (1way ANOVA). Metabolic activity was significantly reduced following FBZ oral 15mg/kg qd x 5 days (Unpaired T-test, *P<0.05) in experiment B.

Fig 2 details the metabolic activity of male *O*. *ochengi* in mice that contained viable, motile worms five weeks post-dosing for both experiments. Motile worms isolated from single injected FBZ treated mice displayed similar metabolic activity compared with untreated controls. Further, motile *O*. *ochengi* recovered from multiple dosed oral FBZ mice (at 0.2 or 1.5mg/kg) did not show a significant reduction in metabolic activity compared with untreated controls. However, in adult motile *O*. *ochengi* derived from mice treated with FBZ orally at 15mg/kg QD x5, the metabolic activity was significantly reduced compared with the matching vehicle controls (unpaired T test, *P =* 0.0496).

### Assessment of *B*. *malayi* microfilaraemias post-dosing with single oral dose FBZ

Table 5 details the change in peripheral circulating microfilaraemias initially recorded at baseline and at two days following single oral dose with the positive control IVM at 0.2mg/kg compared with FBZ at 2 or 40mg/kg. Only IVM mediated a significant, 80.9% rapid reduction in circulating levels of *Bm*mf. In comparison, the change in peripheral circulating mf following low or high dose FBZ were not significantly different to reductions evident in the vehicle control group (11.8% and 49% efficacy, 2 and 40mg/kg, respectively, *vs* 37.5%, vehicle).

**Table 5:** Change in peripheral *B*. *malayi* microfilaraemias two days after single oral FBZ.

group	Geometric mean baseline (95% CI)	Geometric mean +2d (95% CI)	% efficacy +2d
Vehicle	1572 (1000–2471)	982 (672–1435)	37.5
**IVM 0.2mg/kg**^#^	**2041** **(1651–2523)**	**388** **(278–543)**	**80.9**
FBZ 2mg/kg	1284 (1069–1541)	1133 (640–2005)	11.8
FBZ 40mg/kg	1806 (1034–3156)	921 (432–1961)	49.0

^#^1 way ANOVA (F = 5.812, *P =* 0.007) with Dunnett’s tests post-hoc (vehicle *vs* IVM, *P<*0.05).

**Fig 3:**
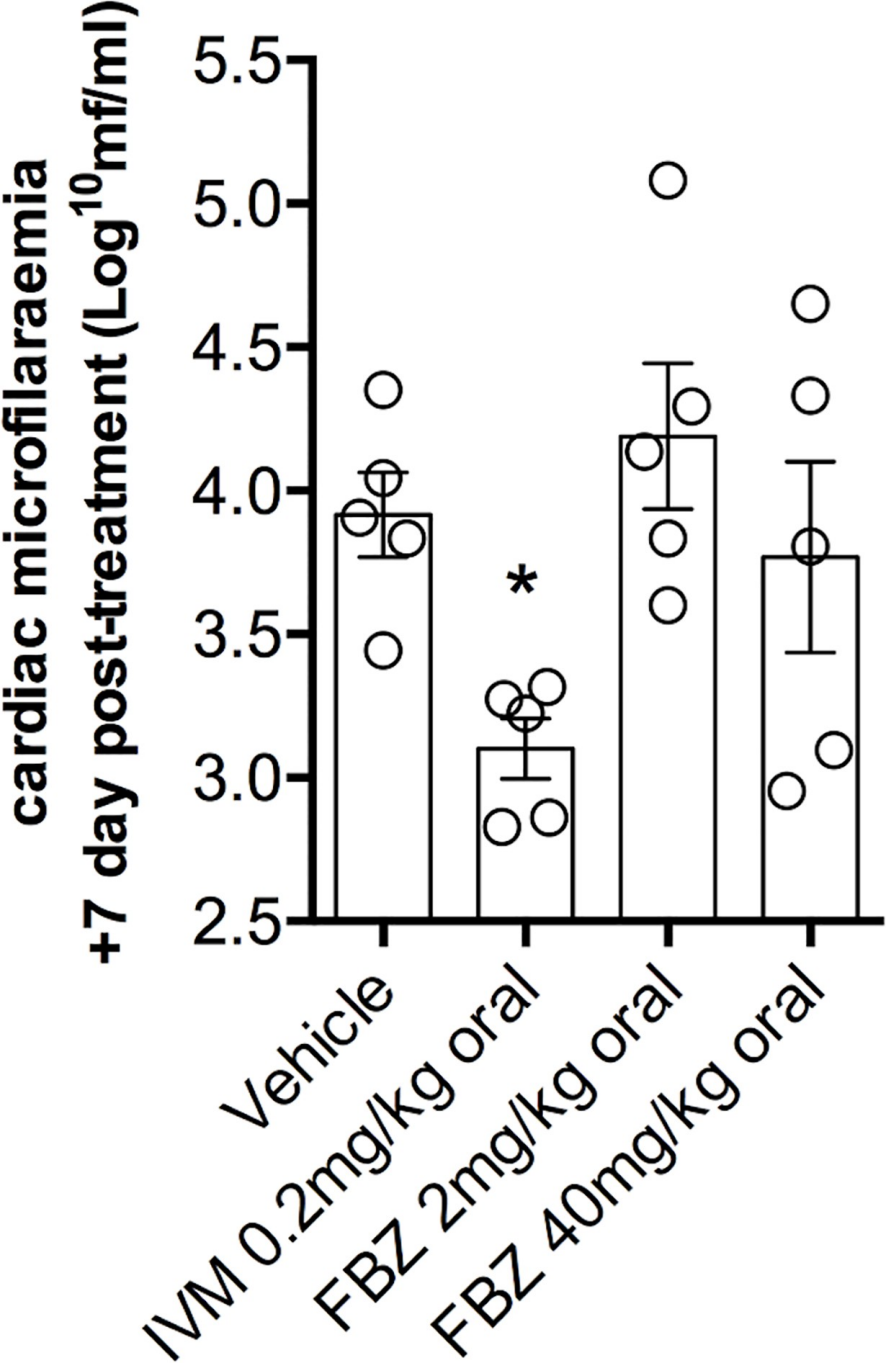
Average *B*. *malayi* microfilariaemias in cardiac blood seven days post-dosing with indicated treatments. Data points are microfilariaemias per mouse. Bars are mean +/-SEM per treatment group. Variation between untreated and treatment groups was significantly different (1way ANOVA F = 4.6, *P =* 0.019). Significant differences compared with vehicle controls, evaluated by Dunnett’s tests, are indicated * (*P*<0.05).

After seven days following treatment, the level of *B*. *malayi* mf in cardiac blood was assessed following necropsy (Fig 3). The geometric mean level of microfilaraemia in vehicle treated animals was 8236mf/ml (985–19486 95% C.I.). Single dose IVM-treated animals had a significantly reduced burden of microfilaraemia (84.7% efficacy, 1way ANOVA F = 4.6, *P =* 0.019, *P*<0.05, vehicle *vs* IVM, Dunnett’s post-hoc test). In comparison the levels of parasitaemias in low or high single doses of FBZ were not significantly reduced compared with vehicle.

### Pharmacokinetic analysis and modelling

For all experiments, the plasma concentrations and pharmacokinetics parameters of FBZ, H-FBZ and R-FBZ are depicted in Table 6. After single subcutaneous administration of FBZ to male SCID mice at 10mg/kg, peak plasma concentrations were observed 1hr after dosing. After multiple oral administration of FBZ to male SCID mice for 5 days, peak plasma concentrations at day 5 were observed at 0.5hr after dosing at 0.2 and 1.5mg/kg, and at 1hr after dosing at 15mg/kg, suggesting a rapid absorption. Cmax and AUClast values increased dose proportionally. In experiment A, the R-FBZ/FBZ ratio ranged from 0.1 to 0.4 and from 0.3 to 0.7 for H-FBZ/FBZ across all FBZ dosed groups. In experiment B, the R-FBZ/FBZ ratio was 0.4, the H-FBZ/FBZ ratio was 0.55.

**Table 6:** FBZ pharmacokinetic parameters following 5 days of dosing.

Group	Day	Treatment	C_max_^|^ (μg/mL)	t_max_^^^ (h)	terminal *t*_*1/2*_^ℵ^ (h)	AUC_last_ ^#^ (μg.h/mL)	H-FBZ /FBZ AUC ratio	R-FBZ/FBZ AUC ratio
A2	**5**	10mg/kg QD 5x SC	0.04	3	648	9.9	0.6	0.2
A3	**1**	10mg/kg QD 1x SC	0.07	1	332	5.3	0.3	0.1
A4	**5**	0.2mg/kg QD 5x OR	0.03	0.5	4	0.12	0.4	0.2
A5	**5**	1.5mg/kg QD 5x OR	0.3	0.5	0.5	0.72	0.7	0.4
B2	**5**	15mg/kg QD 5x OR	2.91	1.0	2	10.3	0.55	0.40
C3	**1**	2mg/kg 1x OR	0.4	0.5	2	1.4	0.5	0.4
C4	**1**	40mg/kg 1x OR	3.7	0.5	3.5	14	0.4	0.6

*A = *O*. *ochengi* adult implant experiment A, B = *O*. *ochengi* adult implant experiment B, C = *B*. *malayi* mf infusion experiment

^**|**^C_max_: highest observed plasma concentration

^t_max_: time point when C_max_ is observed

#AUC_last_: Area Under the plasma concentration *vs*. time curve (_last_: last time point where plasma concentration is measured); H-FBZ: hydrolysed FBZ; R-FBZ: reduced FBZ

ℵ terminal *t*_*1/2*_: Apparent terminal elimination half-life (estimated graphically from simulated PK profiles)

**Fig 4:**
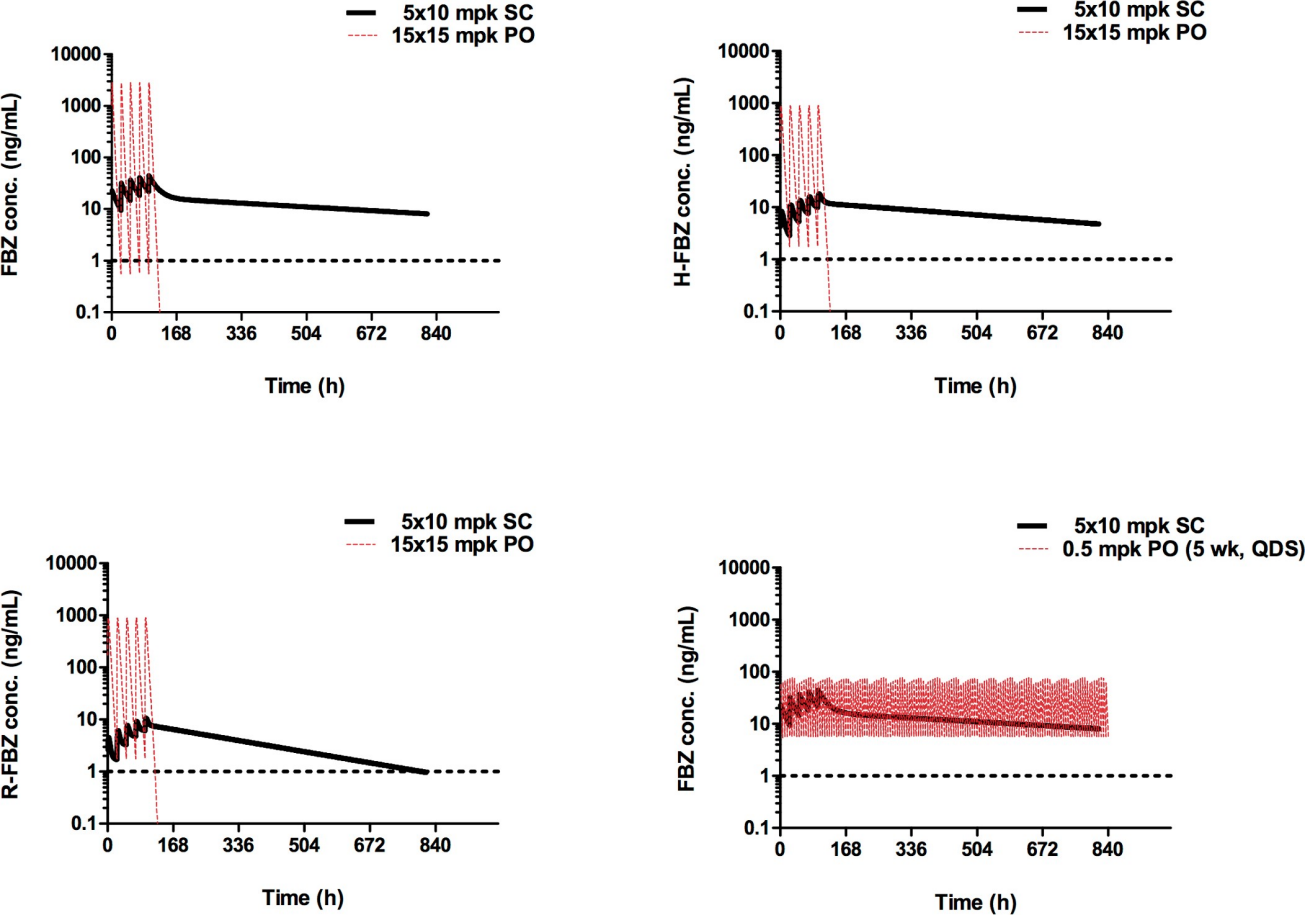
Comparison between oral PK profile (dashed red lines) and s.c. injection PK profile (solid black line) in CB. 17 SCID mice as assessed from PK modelling of exposure data. (**Top left)** FBZ PK after 10mg/kg qd 5x s.c. FBZ or 15mg/kg qd 5x oral FBZ. **(Top right)** H-FBZ PK after 10mg/kg qd 5x s.c. FBZ or 15mg/kg qd 5x oral FBZ. **(bottom left)** R-FBZ PK after 10mg/kg qd 5x s.c. or 15mg/kg qd 5x oral FBZ. **(bottom right)** Simulated FBZ PK profile after 10mg/kg qd 5x s.c. or 5 week, 4 times daily (qds) regimen of 0.5mg/kg oral FBZ.

After single administration of FBZ to male SCID mice at 2 and 40 mg/kg, peak plasma concentrations were observed at 0.5hr after dosing, suggesting a rapid absorption. C_max_ and AUC_inf_ values increased less than dose proportionally to the dose between 2mg/kg/day up to 40mg/kg. In this latter study the R-FBZ/FBZ ratio ranged from 0.4 to 0.6 and from 0.4 to 0.5 for H-FBZ/FBZ across both FBZ dosed groups. The PK exposures of parental FBZ and its major metabolites FBZ-R and FBZ-H at the highest oral dose (15mg/kg QD 5x) or the injected dose (10mg/kg QD 5x) were simulated and compared with one another using a PK modelling approach (Fig 4 & S1 Data). From these simulations, an oral dose regimen that more closely emulated the exposure profile of multiple injected FBZ was determined as 0.2 mg/kg QID for 35 days.

## Discussion

FBZ has been proposed as a relatively ‘low hanging fruit’ to reformulate and repurpose as an oral *Onchocerca* macrofilaricide [24, 39]. Delivered as a multiple injection, FBZ is highly potent in mediating rapid death of *O*. *ochengi* [33] and *O*. *volvulus in vivo* [24, 32]. Additionally, as with other members of the benzimidazole (BZ) class, FBZ is more efficacious at targeting adult *Onchocerca* rather than *Onchocerca* mf [24, 40], making it an attractive option for an indication where rapid microfilaricidal activity would want to be avoided (e.g. areas of *L*. *loa* co-endemicity).

Selective toxicity stems from BZ binding to nematode β tubulin with approximately 10-fold greater affinity than mammalian tubulin, principally due to polymorphisms around amino acid position 200. Efficacy appears to be most readily manifest in embryotoxic effects on female worms, whereby prevention of microtubule elongation interferes with chromosome segregation and mitotic cell division, leading to defective embryogenesis. A fecund female intra-nodular *Onchocerca* small animal model is currently not available to scrutinise this specific impact of oral FBZ, which may be potentially delinked from macrofilaricidal activity (i.e. sterilising activity only). However, surrogate filarial models of onchocerciasis (*Brugia pahangi* and *Litomosoides sigmodontis* gerbil infection models) allow for scrutiny of embryogenesis post-drugging. Efficacy testing with oral FBZ at matching doses has been undertaken in these lymphatic filarial infection models by independent laboratories and their findings will be published elsewhere.

Beyond targeting the female germline, other rapidly dividing filarial cells would also presumably be sensitive to the β tubulin capping and subsequent mitosis blocking effects of FBZ. Indeed, histopathological evidence suggests intestinal and hypodermal tissue abnormalities are rapidly manifest after brief *in vitro* exposure of adult filariae to FBZ [41]. Furthermore, multiple injections target male worms *in vivo* [33].

Three concerns have been raised over the development of FBZ as a macrofilaricide: First, the parenteral route of administration is not compatible with a field-based NTD indication and intra-muscular injections cause undesirable inflammatory adverse reactions at the injection site. Second, FBZ is poorly bioavailable when given orally. The third concern is that FBZ has a narrow safety margin before deleterious effects on mammalian cell division, which may lead to carcinogenic toxicity, are detected in preclinical toxicological assays.

Addressing the first two caveats, we have been successful in developing an orally-bioavailable formulation of FBZ. Sparse PK measurements taken in SCID mice dosed during our infection experiments demonstrate that rapid and dose-proportional absorption occurred after oral dosing with the FBZ Bend 1/9 formulation, confirming previous rich PK measurements. The PK profile of the multiple oral administered FBZ displayed a distinct profile compared with that of subcutaneous injections. Whilst oral dosing mediated upwards of >50 fold higher Cmax of the active FBZ-R than achievable with injections, the injected FBZ gave a sustained chronic exposure of FBZ-R over 35 days of exposure.

We hypothesized that the high Cmax obtained following oral dosing would mitigate against the much-reduced systemic half-life of the active FBZ-R metabolite in mediating macrofilaricidal activity. However, initial *in vivo* testing with 0.2 or 1.5 mg/kg dosing for five days did not achieve any notable adulticidal activity against implanted *O*. *ochengi* male worms (0 & 19% efficacy, respectively) nor was there any significant indication of reduced viability of *O*. *ochengi* retrieved from SCID mice 35 days after dosing. When elevating the dose to 15 mg/kg, a sub-optimal 30% efficacy was recorded with partial yet significant reductions in viability assays of the retrieved *O*. *ochengi* parasites at +35 days. Because filariae with reduced viability following short high dose FBZ exposures (24 hour, 10μM) can recover after washout *in vivo*, the partially reduced metabolic activity detected following 15mg/kg oral treatment may reflect a temporary and reversible drug effect [41]. This was in marked contrast to parenterally-delivered FBZ which achieved 82% macrofilaricidal efficacy after a single injection. Confirming our previous observations [33], multiple injections were still required to deliver >90% efficacy against adult *O*. *ochengi*. Further oral dose elevations beyond 15mg/kg for 5 days were ruled out in this preclinical model due to toxicological findings indicating a negative safety window at or beyond this dose level. Using PK modelling we show that to maintain FBZ levels consistent to those observed in efficacious SC dosing, a 4 time daily dosing of 0.2mg/kg of the oral formulation over 35 days is needed. Such a dosing regime would maintain FBZ levels above those obtained from 10mg/kg QD 5x SC for at least 80% of the whole duration of treatment. The need for multiple dosing per day is due to the oral formulation’s short half-life (~2h). The most likely reason for the longer exposure achieved by SC administration of FBZ is its slow absorption across the subcutaneous barrier which creates a depot effect allowing for the drug to be released steadily over a period of >35 days.

We coincidently tested whether oral FBZ and its metabolites impacted on circulating *B*. *malayi* mf in the blood (as a surrogate bloodborne microfilarial model of *L*. *loa*). The rationale for this was to evaluate any potential safety risk of oral FBZ mediating rapid ‘IVM-like” microfilaricidal activity. Despite the increased peak plasma concentration of the oral formulation, we did not identify any evidence that FBZ was directly microfilaricidal and conclude that elevated exposures of this BZ drug are not likely to mediate substantial rapid activity against bloodborne mf.

In conclusion, oral dosing with the FBZ Bend 1/9 formulation achieves bioavailability of FBZ and its active metabolite but does not confer significant macrofilaricidal activity against adult *O*. *ochengi* nor significant microfilaricidal activity against bloodborne mf in the pan-filarial SCID mouse *in vivo* models utilised. Efficacy is not driven by *C*_*max*_ but rather by sustained drug levels over long periods of time as indicated by the discrepancy between the terminal half-life of FBZ when administered subcutaneously (apparent terminal *t*_*1/2*_ up to 648h) and when administered orally (apparent terminal *t*_*1/2*_ 0.5h-4h). This discrepancy can be further appreciated by comparing the simulated exposure profiles of FBZ when administered orally and subcutaneously at similar doses. Evidently, the markedly higher initial levels of drug in the oral formulations (~70-fold higher *C*_*max*_) are redundant in terms of producing superior pharmacological activity. A prolonged exposure lasting for ~35 days however, even at peak concentrations that are ~70-fold lower than what is observed in current oral profiles could achieve better macrofilaricidal efficacy. Either a sustained release formulation or prolonged oral dosing durations would be necessary to achieve matching efficacious exposure to injection routes but caution in this approach would be necessary given the low safety window determined for this drug.

## Supporting information

S1 Data Results of compartmental PK analysis as determined from PK modelling of FBZ exposure data. (DOCX)

## References

[1] Meeting of the International Task Force for Disease Eradication, January 2014. Wkly Epidemiol Rec. 2014;89(15):153–60. 24754045

[2] Bockarie MJ, Kelly-Hope LA, Rebollo M, Molyneux DH. Preventive chemotherapy as a strategy for elimination of neglected tropical parasitic diseases: endgame challenges. Philos Trans R Soc Lond B Biol Sci. 2013;368(1623):20120144 10.1098/rstb.2012.0144 23798692 PMC3720042

[3] Taylor MJ, Hoerauf A, Bockarie M. Lymphatic filariasis and onchocerciasis. Lancet. 2010;376(9747):1175–85. 10.1016/S0140-6736(10)60586-7 20739055

[4] [APOC] APfOC. Final communiqué Joint Action Forum (JAF) of APOC; Paris, France2005.

[5] WHO. Onchocerciasis Factsheet No 374. 2016.

[6] WHO. Onchocerciasis and its control: Report of a WHO Expert Committee on Onchocerciasis Control. 1995. 7541171

[7] African Programme for Onchocerciasis Control—report of the sixth meeting of National Task Forces, October 2009. Wkly Epidemiol Rec. 2010;85(4):23–8. 20095110

[8] Turner JD, Langley RS, Johnston KL, Gentil K, Ford L, Wu B, et al Wolbachia lipoprotein stimulates innate and adaptive immunity through Toll-like receptors 2 and 6 to induce disease manifestations of filariasis. J Biol Chem. 2009;284(33):22364–78. 10.1074/jbc.M901528200 19458089 PMC2755959

[9] Pearlman E, Hall LR, Higgins AW, Bardenstein DS, Diaconu E, Hazlett FE, et al The role of eosinophils and neutrophils in helminth-induced keratitis. Invest Ophthalmol Vis Sci. 1998;39(7):1176–82. 9620077

[10] Remme JHF, Feenstra P, Lever PR, Medici AC, Morel CM, Noma M, et al. Tropical Diseases Targeted for Elimination: Chagas Disease, Lymphatic Filariasis, Onchocerciasis, and Leprosy. In: Jamison DT, Breman JG, Measham AR, Alleyne G, Claeson M, Evans DB, et al., editors. Disease Control Priorities in Developing Countries. 2nd ed. Washington (DC)2006. 21250324

[11] WHO. Global Plan to Combat Neglected Tropical Diseases 2008–2017 2007 [Available from: http://apps.who.int/iris/bitstream/10665/69708/1/WHO_CDS_NTD_2007.3_eng.pdf.

[12] WHO. Accelerating Work to Overcome the Global Impact of Neglected Tropical Diseases: A Roadmap for Implementation 2012 [Available from: http://www.who.int/neglected_diseases/NTD_RoadMap_2012_Fullversion.pdf.

[13] Dunn C, Callahan K, Katabarwa M, Richards F, Hopkins D, Withers PC Jr., et al The Contributions of Onchocerciasis Control and Elimination Programs toward the Achievement of the Millennium Development Goals. PLoS Negl Trop Dis. 2015;9(5):e0003703 10.1371/journal.pntd.0003703 25996946 PMC4440802

[14] Molyneux DH, Bradley M, Hoerauf A, Kyelem D, Taylor MJ. Mass drug treatment for lymphatic filariasis and onchocerciasis. Trends Parasitol. 2003;19(11):516–22. 14580963 10.1016/j.pt.2003.09.004

[15] Cupp EW, Sauerbrey M, Richards F. Elimination of human onchocerciasis: history of progress and current feasibility using ivermectin (Mectizan((R))) monotherapy. Acta Trop. 2011;120 Suppl 1:S100–8. 20801094 10.1016/j.actatropica.2010.08.009

[16] Basanez MG, Pion SD, Churcher TS, Breitling LP, Little MP, Boussinesq M. River blindness: a success story under threat? PLoS Med. 2006;3(9):e371 10.1371/journal.pmed.0030371 17002504 PMC1576321

[17] Evans DS, Alphonsus K, Umaru J, Eigege A, Miri E, Mafuyai H, et al Status of Onchocerciasis transmission after more than a decade of mass drug administration for onchocerciasis and lymphatic filariasis elimination in central Nigeria: challenges in coordinating the stop MDA decision. PLoS Negl Trop Dis. 2014;8(9):e3113 10.1371/journal.pntd.0003113 25233351 PMC4169246

[18] Bourguinat C, Pion SD, Kamgno J, Gardon J, Duke BO, Boussinesq M, et al Genetic selection of low fertile Onchocerca volvulus by ivermectin treatment. PLoS Negl Trop Dis. 2007;1(1):e72 10.1371/journal.pntd.0000072 17989786 PMC2041821

[19] Osei-Atweneboana MY, Awadzi K, Attah SK, Boakye DA, Gyapong JO, Prichard RK. Phenotypic evidence of emerging ivermectin resistance in Onchocerca volvulus. PLoS Negl Trop Dis. 2011;5(3):e998 10.1371/journal.pntd.0000998 21468315 PMC3066159

[20] Osei-Atweneboana MY, Boakye DA, Awadzi K, Gyapong JO, Prichard RK. Genotypic analysis of beta-tubulin in Onchocerca volvulus from communities and individuals showing poor parasitological response to ivermectin treatment. Int J Parasitol Drugs Drug Resist. 2012;2:20–8. 10.1016/j.ijpddr.2012.01.005 24533268 PMC3862422

[21] Taylor MJ, Awadzi K, Basanez MG, Biritwum N, Boakye D, Boatin B, et al Onchocerciasis Control: Vision for the Future from a Ghanian perspective. Parasit Vectors. 2009;2(1):7 10.1186/1756-3305-2-7 19154624 PMC2639371

[22] Gardon J, Gardon-Wendel N, Demanga N, Kamgno J, Chippaux JP, Boussinesq M. Serious reactions after mass treatment of onchocerciasis with ivermectin in an area endemic for Loa loa infection. Lancet. 1997;350(9070):18–22. 10.1016/S0140-6736(96)11094-1 9217715

[23] Taylor MJ, Hoerauf A, Townson S, Slatko BE, Ward SA. Anti-Wolbachia drug discovery and development: safe macrofilaricides for onchocerciasis and lymphatic filariasis. Parasitology. 2014;141(1):119–27. 10.1017/S0031182013001108 23866958 PMC3884836

[24] Mackenzie CD, Geary TG. Flubendazole: a candidate macrofilaricide for lymphatic filariasis and onchocerciasis field programs. Expert Rev Anti Infect Ther. 2011;9(5):497–501. 10.1586/eri.11.30 21609260

[25] Lacey E. The role of the cytoskeletal protein, tubulin, in the mode of action and mechanism of drug resistance to benzimidazoles. Int J Parasitol. 1988;18(7):885–936. 3066771 10.1016/0020-7519(88)90175-0

[26] Bradley RE, Guerrero J, Becker HN, Michael BF, Newcomb K. Flubendazole: dose range and efficacy studies against common internal parasites of swine. Am J Vet Res. 1983;44(7):1329–33. 6881670

[27] Horton RJ. Benzimidazoles in a wormy world. Parasitol Today. 1990;6(4):106 15463310 10.1016/0169-4758(90)90225-s

[28] Kan SP. The anthelmintic effects of flubendazole on Trichuris trichiura and Ascaris lumbricoides. Trans R Soc Trop Med Hyg. 1983;77(5):668–70. 6659046 10.1016/0035-9203(83)90199-2

[29] Yangco BG, Klein TW, Deresinski SC, Vickery AC, Craig CP. Flubendazole and mebendazole in the treatment of trichuriasis and other helminthiases. Clin Ther. 1981;4(4):285–90. 7332916

[30] Denham DA, Samad R, Cho SY, Suswillo RR, Skippins SC. The anthelmintic effects of flubendazole on Brugia pahangi. Trans R Soc Trop Med Hyg. 1979;73(6):673–6. 538808 10.1016/0035-9203(79)90018-x

[31] Mak JW. Antifilarial activity of mebendazole and flubendazole on Breinlia booliati. Trans R Soc Trop Med Hyg. 1981;75(2):306–7. 7303142 10.1016/0035-9203(81)90343-6

[32] Dominguez-Vazquez A, Taylor HR, Greene BM, Ruvalcaba-Macias AM, Rivas-Alcala AR, Murphy RP, et al Comparison of flubendazole and diethylcarbamazine in treatment of onchocerciasis. Lancet. 1983;1(8317):139–43. 6130195 10.1016/s0140-6736(83)92753-8

[33] Halliday A, Guimaraes AF, Tyrer HE, Metuge HM, Patrick CN, Arnaud KO, et al A murine macrofilaricide pre-clinical screening model for onchocerciasis and lymphatic filariasis. Parasit Vectors. 2014;7:472 10.1186/s13071-014-0472-z 25338621 PMC4212127

[34] Ceballos L, Mackenzie C, Geary T, Alvarez L, Lanusse C. Exploring the potential of flubendazole in filariasis control: evaluation of the systemic exposure for different pharmaceutical preparations. PLoS Negl Trop Dis. 2014;8(5):e2838 10.1371/journal.pntd.0002838 24874646 PMC4038472

[35] Longo M, Zanoncelli S, Messina M, Scandale I, Mackenzie C, Geary T, et al In vivo preliminary investigations of the effects of the benzimidazole anthelmintic drug flubendazole on rat embryos and fetuses. Reprod Toxicol. 2014;49:33–42. 10.1016/j.reprotox.2014.06.009 24994687

[36] Aljayyoussi G, Tyrer HE, Ford L, Sjoberg H, Pionnier N, Waterhouse D, et al Short-Course, High-Dose Rifampicin Achieves Wolbachia Depletion Predictive of Curative Outcomes in Preclinical Models of Lymphatic Filariasis and Onchocerciasis. Sci Rep. 2017;7(1):210 10.1038/s41598-017-00322-5 28303006 PMC5428297

[37] Shapiro SS, Wilk MB. An analysis of variance test for normality (complete samples). Biometrika. 1965;52(3–4):591–611.

[38] Dunn JD. Multiple Comparisons Using Rank Sums. Technometrics. 1964;6(3):241–52.

[39] Geary TG, Mackenzie CD. Progress and challenges in the discovery of macrofilaricidal drugs. Expert Rev Anti Infect Ther. 2011;9(8):681–95. 10.1586/eri.11.76 21819332

[40] Townson S, Dobinson A, Connelly C, Muller R. Chemotherapy of Onchocerca lienalis microfilariae in mice: a model for the evaluation of novel compounds for the treatment of onchocerciasis. J Helminthol. 1988;62(3):181–94. 3192910 10.1017/s0022149x00011494

[41] O'Neill M, Geary JF, Agnew DW, Mackenzie CD, Geary TG. In vitro flubendazole-induced damage to vital tissues in adult females of the filarial nematode Brugia malayi. Int J Parasitol Drugs Drug Resist. 2015;5(3):135–40. 10.1016/j.ijpddr.2015.06.002 26288741 PMC4534755

