# Supplementary material for: Short-course, oral flubendazole does not mediate significant efficacy against Onchocerca adult male worms or Brugia microfilariae in murine infection models
Source: PLoS Negl Trop Dis. 2019 Jan 16;13(1):e0006356. doi: 10.1371/journal.pntd.0006356 (PMC6334903; doi:10.1371/journal.pntd.0006356)
Supplement: S1 Data — (DOCX) [file pntd.0006356.s001.docx]

S1 Data

***PK modelling and simulation***

For PK analysis Subcutaneous and Oral data were fitted using the following equations.

$$\frac{dX_{1}}{dt}= -k_{a}\cdot X_{1}$$

$$\frac{dX_{2}}{dt}= k_{a}\cdot X_{1}-\left( k_{cp}+k_{e} \right)\cdot X_{2}+ k_{pc}\cdot X_{3}$$

$$\frac{dX_{3}}{dt}= k_{cp}\cdot X_{2}-k_{pc}\cdot X_{3}$$

$$C= \frac{X_{2}}{V}$$

Where ***X_1_*** represents the drug mass in dosing compartment, ***X_2_*** represents drug mass in the systemic circulation and ***X_3_*** represents the drug mass peripheral compartment. ***k_a_*** represents the rate of absorption (*h^-1^*), ***k_e_*** the rate of drug elimination (*h^-1^*), ***k_cp_*** and ***k_pc_*** the rates of transfer between the central and peripheral compartments (*h^-1^*), ***V*** represents the volume of distribution (*mL*) and ***C*** is the concentration of drug at any given time (*mg/L*).

For the oral preparations, a one compartment model was sufficient to fit the data as evidenced by AIC and BIC parameters as well as the intercompartmental transfer rates being negligible. For these reasons, the parameters *k_cp_* and *k_pc_* were fixed to zero to fit the oral data into a one compartment model.

Results of compartmental PK analysis as determined from PK modelling of FBZ exposure data.

|  |  | **PK Parameters (FBZ)** | | | | |
| --- | --- | --- | --- | --- | --- | --- |
|  | **Dosing Regime** | ***k_a_*** *(h^-1^)* | ***k_e_*** *(h^-1^)* | ***k_cp_*** *(h^-1^)* | ***k_pc_*** *(h^-1^)* | ***V/F*** *(L/kg)* |
| *O.ochengi* male implants | 5x10 mg/kg SC | 5.71 | 0.0089 | 0.062 | 0.021 | 260.4 |
|  | 1x10 mg/kg SC | 9.81 | 0.006 | 0.036 | 0.0082 | 430.0 |
|  | 5x0.2 mg/kg PO | 9.0 | 0.47 | N/A | N/A | 5.9 |
|  | 5x1.5 mg/kg PO | 3.26 | 0.65 | N/A | N/A | 3.5 |
|  | 5x15 mg/kg PO | 2.58 | 0.37 | N/A | N/A | 3.9 |
| *B. malayi* mf infusions | 1x2mg/kg PO | 15.0 | 0.44 | N/A | N/A | 3.8 |
|  | 1x40mg/kg PO | 15.0 | 0.28 | N/A | N/A | 9.6 |
